# Bioinformatics identification of CCL8/21 as potential prognostic biomarkers in breast cancer microenvironment

**DOI:** 10.1042/BSR20202042

**Published:** 2020-11-24

**Authors:** Bowen Chen, Shuyuan Zhang, Qiuyu Li, Shiting Wu, Han He, Jinbo Huang

**Affiliations:** 1 Department of Breast Disease, Maoming People’s Hospital, Maoming 525000, China; 2 Department of Clinical Laboratory, Maoming People’s Hospital, Maoming 525000, China; 3 Department of Emergency, Maoming People’s Hospital, Maoming 525000, China; 4 Department of Oncology, Maoming People’s Hospital, Maoming 525000, China; 5 Department of Medical Imaging, Maoming People’s Hospital, Maoming 525000, China

**Keywords:** biomarker, breast cancer, CCL21, CCL8, tumor microenvironments

## Abstract

***Background:*** Breast cancer (BC) is the most common malignancy among females worldwide. The tumor microenvironment usually prevents effective lymphocyte activation and infiltration, and suppresses infiltrating effector cells, leading to a failure of the host to reject the tumor. CC chemokines play a significant role in inflammation and infection.

***Methods:*** In our study, we analyzed the expression and survival data of CC chemokines in patients with BC using several bioinformatics analyses tools.

***Results:*** The mRNA expression of CCL2/3/4/5/7/8/11/17/19/20/22 was remarkably increased while CCL14/21/23/28 was significantly down-regulated in BC tissues compared with normal tissues. Methylation could down-regulate expression of CCL2/5/15/17/19/20/22/23/24/25/26/27 in BC. Low expression of CCL3/4/23 was found to be associated with drug resistance in BC. Results from Kaplan–Meier plotter and BC Gene-Expression Miner v4.2 (bcGenExMiner) v4.2 demonstrated that BC patients with high CCL8 and low CCL19/21/22 expression were more likely to have a worse prognosis. CCL8 expression was significantly up-regulated in BC tissues compared with normal tissues. High CCL8 expression was significantly correlated with negative PR, negative ER, positive nodal status, triple-negative BC subtype, basal-like BC subtype, triple-negative and basal-like BC subtype and high grades. CCL21 was down-regulated in BC, while high levels of CCL21 was associated with negative PR, triple-negative subtype, basal-like subtype and low tumor grade. Functional analysis demonstrated that CCL8 and CCL21 were involved in carcinogenesis, tumor immune escape and chemoresistance in BC.

***Conclusion:*** Integrative bioinformatics analysis demonstrated CCL8/21 as potential prognostic biomarkers in BC microenvironment.

## Background

Breast cancer (BC), the most common malignancy among females worldwide, is diagnosed in 2.1 million women each year and constitutes almost a quarter of all cancer cases among women [1]. Though notable steps have been taken towards early detection and treatment of BC and survival rates have undergone significant improvement, long-term survival is as disappointing as before and the prognosis of BC patients in the advanced stage or with metastasis remains poor [2]. Previous studies have revealed that estrogen (ER), progesterone (PR), and epidermal growth factor 2 (HER2) status are critical in determining the treatment regimen for BC patients [3]. The tumor microenvironment, which is composed of tumor cells, stromal cells, inflammatory cells, vasculature and extracellular matrices, usually prevents effective lymphocyte activation and infiltration, and suppresses infiltrating effector cells, leading to a failure of the host to reject the tumor [4]. Increasing evidence demonstrates that immune system disorders are closely related to the occurrence and progression of tumors, including BC [5]. Therefore, it is urgent and significant to identify novel immune biomarkers for BC in order to develop individualized treatment plans for patients.

Chemokines, small secreted signaling proteins, have been identified as important mediators of inflammatory responses and modulators of immune cell trafficking via interaction with chemokine receptors [6]. Chemokines fused to anti-tumor antibodies can help attract adoptive metastatic tumor antigen (Ag)-specific T cells to the tumor site [7]. There are four main classes of chemokines: the CXC chemokines, the CC chemokines, the C chemokines and CXC chemokines. Several studies have revealed that chemokines secreted from tumor cells play a stimulated role in tumor growth, progression and metastasis [8]. Additionally, the use of chemokines as biomarkers for prognostic prediction of cancers, including BC, has attracted the attention of increasingly more researchers [9,10].

Overall, 28 CC (CCL1–28) chemokines have been identified in humans. As the largest family of chemokines, CC chemokines play a significant role in inflammation and infection [11]. With a mass of 8–14 kDa, CC chemokines could defend chemokines against proteolytic degradation with highly diverse quaternary structures and can therefore modulate diverse immune functions [12]. Increasing results have emphasized the potential of CC chemokines in the tumor microenvironment as prognostic biomarkers and therapeutic targets across a variety of tumors [13,14]. Previous studies have revealed that CC chemokines are involved in tumorigenesis and progression of BC, and thus have an effect on the prognosis of BC patients [15,16]. However, these studies were performed in a small cohort with certain limitations. Furthermore, these studies only clarified the role of specific CC chemokines in invasive BC (BRCA), rather than all additional CC chemokines. In our study, we explore the expression, prognosis and associated function of all the CC chemokines in invasive BC via integrative bioinformatics analysis.

## Materials and methods

### ONCOMINE analysis

A publicly accessible web-based cancer microarray database, ONCOMINE (www.oncomine.org) was utilized to facilitate analyses of genome-wide expression. The mRNA levels of CC chemokines in invasive BC were analyzed using ONCOMINE [17]. Student’s *t* test was utilized to evaluate differences of transcriptional expression of CC chemokines among BC samples and normal control. The thresholds were demarcated as: *P*-value = 0.01, fold-change = 2, Gene rank = Top 10% and data type = mRNA.

### GEPIA dataset analysis

GEPIA (http://gepia.cancer-pku.cn/index.html) is an interactive web server that includes RNA sequencing expression data from 9736 tumors and 8587 normal samples [18]. We explored the mRNA expression of CC chemokines in invasive BC tissues and normal breast tissues using the TCGA BRCA dataset (*n*=1085). *P*-value of 0.05 was set as the cutoff.

### GSCALite

GSCALite (http://bioinfo.life.hust.edu.cn/web/GSCALite/) is an online tool that can be utilized to identify the contribution to cancer initiation, progress, diagnosis, prognosis and therapy [19]. GSCALite was used the analyze the methylation and drug sensitivity of CC chemokines using the TCGA BRCA dataset (*n*=1085). Student’s *t* test was performed to identify methylation difference between tumor and normal samples. The *P-*value was adjusted by false discovery rate (FDR). FDR < 0.05 was considered significant. We tested the relationship between paired mRNA expression and methylation, based on Pearson’s product moment correlation coefficient, which followed a T distribution. *P*-value was adjusted by FDR and FDR < 0.05 was set as the threshold. We were then able to obtain genes whose expression was significantly influenced by genomic methylation.

### The Human Protein Atlas

The Human Protein Atlas (https://www.proteinatlas.org) is a map of human proteins present in the cells, tissues and organs identified using integration of various omics technologies (antibody-based imaging, mass spectrometry-based proteomics, transcriptomics and systems biology). In the present study, the ‘tissue module’ and ‘pathology module’ of the Human Protein Atlas was utilized to examine the protein expression of CC chemokines among invasive BC and normal controls [20,21].

### BC Gene-Expression Miner v4.2 v4.2

BC Gene-Expression Miner v4.2 (bcGenExMiner v4.2, http://bcgenex.centregauducheau.fr), an easy practical online platform, analyzes gene prognosis in BC and can be mainly applied to three classical mining functional analyses (expression, prognosis and correlation) based on 36 noted genomic datasets of 5696 patients [22,23]. The data used in our study was updated on 01/09/2019. The correlation between CC chemokine expression and clinical parameters such as age, nodal status, and receptor statuses and clinical outcome of invasive BC was performed using bcGenExMiner v4.2. These analyses were performed with DNA microarrays’ cohorts of invasive BC (*n*=5696). The medium expression of CC chemokines was set as splitting criterion for high-risk and low-risk groups.

### Kaplan–Meier plotter

Kaplan–Meier plotter (www.kmplot.com) is an online database that contains gene expression and survival data of Gene Expression Omnibus and TCGA. The relapse-free survival (RFS) data of 3951 BC patients and overall survival (OS) data of 1402 BC patients was applied to evaluate the prognostic value of CC chemokines among BC patients [24]. The RFS and OS of patients with BC were grouped into high and low expression groups with median gene expression of CC chemokines as a cut-off point. The prognostic value of CC chemokines was evaluated using a Kaplan–Meier survival plot. *P*<0.05 was considered statistically significant.

### cBioPortal

The cBioPortal (www.cbioportal.org) is a web resource that provides information for the integrative analysis of multiple cancer genes based on TCGA data [25,26]. The frequency of CC chemokines gene alterations, co-expression and network analyses in BC were explored using cBioPortal with mRNA and protein level z-scores of ±2.0. These analyses were performed using TCGA BRCA dataset (*n*=1085).

### GeneMANIA

GeneMANIA (http://www.genemania.org), a well-maintained, user-friendly web server, was utilized for gene prioritization and predicting gene function [27]. In our study, protein–protein interaction network of CC chemokines was obtained via GeneMANIA.

### Functional enrichment analysis

Two publicly accessible and flexible gene-list analysis online web interfaces, Metascape (http://metascape.org) and DAVID 6.8 (https://david.ncifcrf.gov/home.jsp), were applied to gene annotation and analysis [28,29]. In this current study, CC chemokines and neighboring genes that were remarkably related to CC chemokine alterations were subjected to Metascape and DAVID 6.8 for pathway and process enrichment analysis. The Gene Ontology (GO) term (biological process, cellular component, molecular function) and Kyoto Encyclopedia of Genes and Genomes (KEGG) pathways were processed using Metascape and DAVID 6.8. In Metascape, only terms with a *P*-value <0.01, a minimum count of 3, and an enrichment factor > 1.5 were collected and grouped into clusters based on membership similarities with a similarity of >0.3 as a cluster. The results of DAVID 6.8 were then plotted by R project with a *P*-value <0.01 and FDR < 0.05.

### TIMER

TIMER (https://cistrome.shinyapps.io/timer/) is a web server that provides systematic analysis of the abundance of immune infiltrates across diverse cancer types [30]. The correlation between CCL8/CCL21 and immune cells (B cells, CD4^+^ T cells, CD8^+^ T cells, neutrophils, macrophages and dendritic cells) was analyzed with Spearman correlation analysis using the TCGA BRCA dataset (*n*=1085) in TIMER. *P*-value of 0.05 was set as the cutoff.

## Results

### Dysregulated expression of CC chemokines in patients with BC

**Figure 1:**
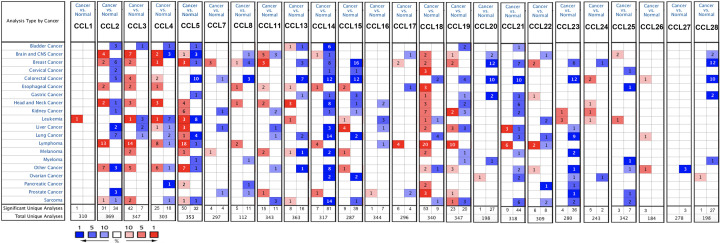
mRNA levels of CC chemokines in BC with the threshold of *P*-value ≤0.01, fold change ≥ 2, and Gene Rank ≥ Top 10% (ONCOMINE) The figure shows the numbers of datasets with statistically significant mRNA overexpression (red) or down-regulated expression (blue) of CC chemokines.

With the exception of CCL6, CCL9, CCL10, and CCL12, a total of 24 CC chemokines were identified in the ONCOMINE database. We compared CC chemokine expression across 20 types of cancers and normal tissues via the ONCOMINE database (Figure 1), which indicated that the mRNA expression of CCL2/3/4/5/7/8/11/17/19/20/22 was significantly up-regulated (Supplementary Figure S1), while CCL14/15/21/23/28 was significantly down-regulated in BC tissues compared with normal tissues (Supplementary Figure S2). Furthermore, data from the GEPIA database indicated increased expression of CCL5/11 and decreased expression of CCL14/21/23/28 in BC tissues (Supplementary Figure S3). Moreover, protein expression data of three CC chemokines (CCL2/4/27) in tumor and normal tissue were acquired from The Human Protein Atlas, which revealed that the protein expression of CCL2/4/27 (Supplementary Figure S4) was remarkably increased in BC tissues. It is worth mentioning that there is not enough protein expression data of the other CC chemokines in the Human Protein Atlas. We also compared expression of 24 CC chemokines in tumor tissues, which revealed that CCL2 was highest expressed in BC tissues (Supplementary Figure S5). In order to identify more CC chemokines associated with the tumorigenesis, progression and clinical outcomes in the BC, we selected all different genes for further study.

### Methylation and drug sensitivity analysis of CC chemokine in BC

**Figure 2:**
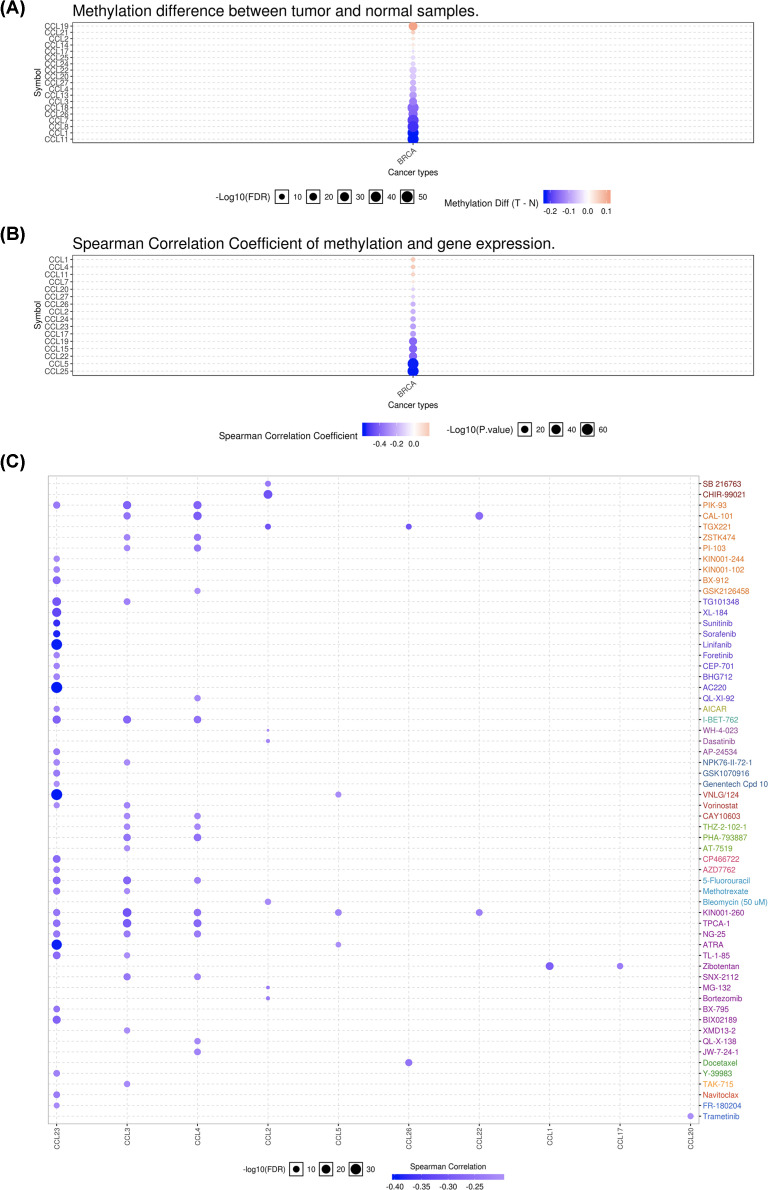
Methylation and drug sensitivity analysis of CC chemokine in BC (**A,B**) Methylation analysis of CC chemokine in BC. (**C**) Drug sensitivity analysis of CC chemokine in BC.

We investigated differential methylation status between BC and normal tissues, which indicated that a total of 19 CC chemokines had significantly differential methylation levels (Figure 2A). Moreover, methylation could down-regulate the level of CCL2/5/15/17/19/20/22/23/24/25/26/27 in BC (Figure 2B). The correlation between CC chemokine expression and drug sensitivity suggests that low expression of CCL3/4/23 are associated with drug resistance (Figure 2C).

### The prognostic value of CC chemokines in patients with BC

**Figure 3:**
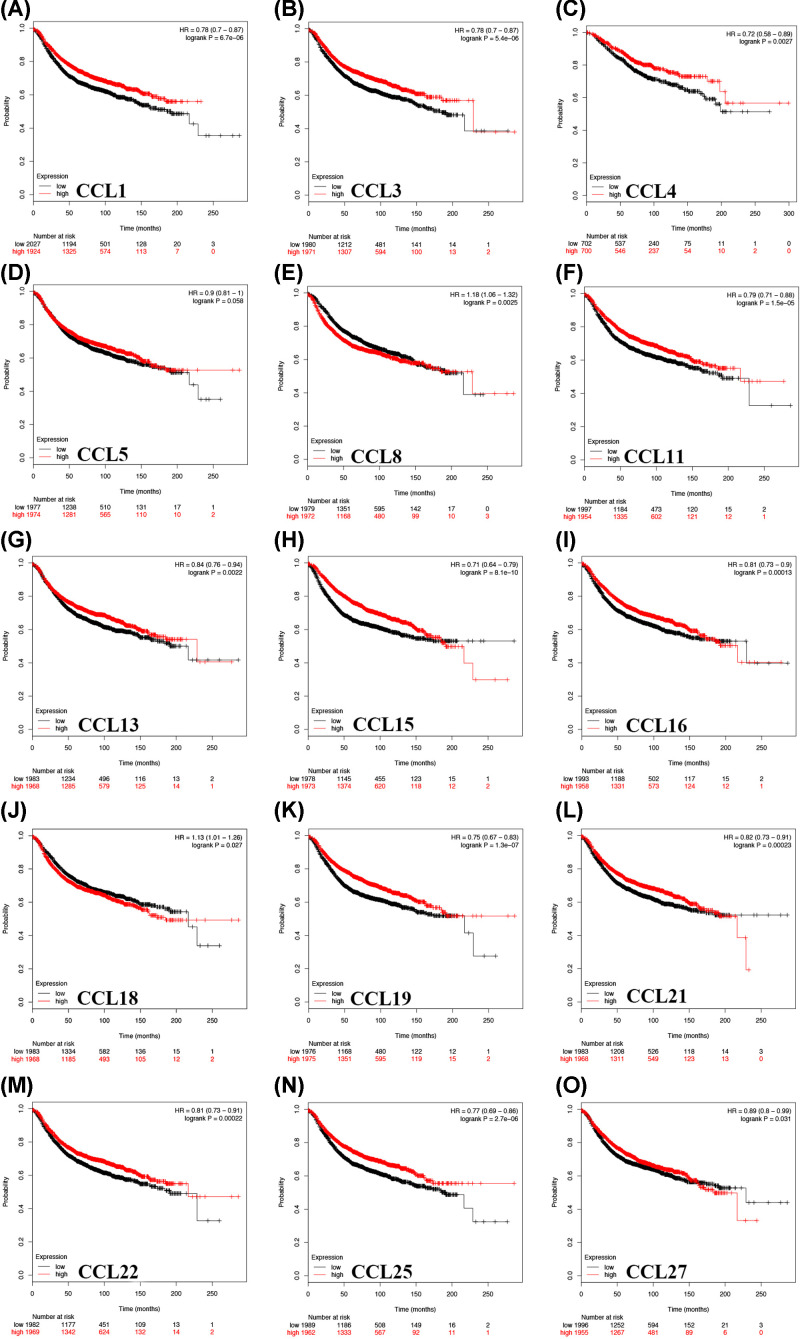
The prognostic value of CC chemokines in BC patients in the RFS curve (Kaplan–Meier plotter) BC patients with high CCL8 (**E**), CCL18 (**J**) mRNA levels and low CCL1 (**A**), CCL3 (**B**),CCL4 (**C**), CCL5 (**D**), CCL11 (**F**), CCL13 (**G**), CCL15 (**H**), CCL16 (**I**), CCL19 (**K**), CCL21 (**L**), CCL22 (**M**), CCL25 (**N**), CCL27 (**O**) mRNA levels were significantly relevant to worse RFS.

**Figure 4:**
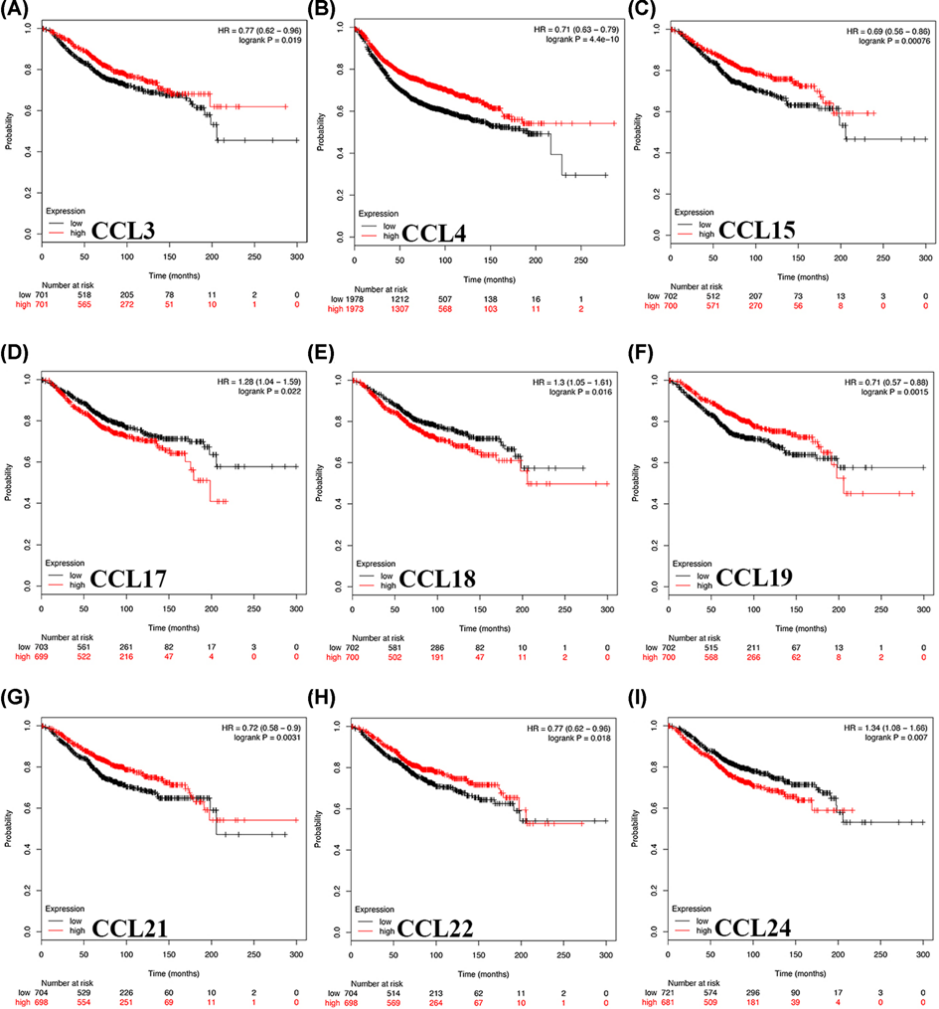
The prognostic value of CC chemokines in BC patients in the OS curve (Kaplan–Meier plotter) BC patients with high CCL17 (**D**), CCL18 (**E**), CCL24 (**F**) mRNA levels and low CCL3 (**A**), CCL4 (**B**), CCL15 (**C**), CCL24 (**I**), CCL21 (**G**), CCL22 (**H**) mRNA levels were significantly relevant to worse OS.

The prognostic value of CC chemokines in patients with BC was evaluated using bcGenExMiner v4.2 and Kaplan–Meier plotter. BC patients were separated into either the high-level group or the low-level group with median expression of CC chemokines of all samples as the cut-off point. Survival data from Kaplan–Meier plotter demonstrated that BC patients with high CCL8/18 mRNA levels and low CCL1/3/4/5/11/13/15/16/19/21/22/25/27 mRNA levels were significantly associated with worse RFS (Figure 3, Table 1; all *P*<0.05, except for CCL5 *P*=0.058). Moreover, survival data from the Kaplan–Meier plotter demonstrated that BC patients with high CCL17/18/24 mRNA levels and low CCL3/4/15/19/21/22 mRNA levels were significantly associated with worse OS (Figure 4, Table 2, all *P*<0.05). Furthermore, we utilized bcGenExMiner v4.2 to further verify our results, which revealed that BC patients with high CCL8 and low CCL19/21/22 expression were significantly more likely to have worse prognosis (Table 3, all *P*<0.05). The intersection results of aforementioned databases demonstrate that CCL8/19/21/22 are associated with clinical outcomes and may exert significant functions in the tumorigenesis and progression of BC (Supplementary Figure S6). However, mRNA expression of CCL19/22 was remarkably increased in BC tissues while the survival data revealed that BC patients with low CCL19/22 expression were significantly more likely to have a worse prognosis. The data from CCL19/22 expression is contrary to the role of CCL19/22 in the prognosis. Therefore, CCL8/21 were selected as targets for further study.

**Table 1:** Prognostic value of CC chemokines expression in BC (bc-GenExMiner v4.2)

Chemokine	Event	*P*-value	HR	95% CI
CCL1	MR	0.2212	0.92	0.81–1.05
CCL2	MR	0.2187	1.08	0.96–1.22
CCL3	MR	0.9834	1	0.82–1.22
CCL4	MR	0.1963	0.92	0.81–1.04
CCL5	MR	0.1908	0.92	0.82–1.04
CCL7	MR	0.1926	1.09	0.96–1.24
CCL8	MR	0.0018	1.23	1.08–1.39
CCL11	MR	0.8628	0.99	0.87–1.12
CCL13	MR	0.1432	1.00	0.97–1.25
CCL14	MR	0.8892	0.97	0.65–1.46
CCL15	MR	0.1885	1.29	0.88–1.89
CCL16	MR	0.0740	0.89	0.79–1.01
CCL17	MR	0.5476	1.04	0.92–1.18
CCL18	MR	0.3420	1.06	0.94–1.20
CCL19	MR	0.0015	0.82	0.72–0.93
CCL20	MR	0.2311	1.08	0.95–1.22
CCL21	MR	0.0006	0.81	0.71–0.91
CCL22	MR	0.0001	0.77	0.67–0.87
CCL23	MR	0.0056	0.83	0.730–0.95
CCL24	MR	0.6538	0.97	0.85–1.10
CCL25	MR	0.1051	1.11	0.98–1.26
CCL26	MR	0.4829	1.06	0.89–1.27
CCL27	MR	0.4840	0.96	0.84–1.09
CCL28	MR	0.8222	0.98	0.79–1.21

Event: metastatic recurrence. Abbreviations: HR, hazard ratio; MR, metastatic recurrence.

**Table 2:** Prognostic value of CC chemokines expression in BC (Kaplan–Meier plotter)

Chemokine	Cut-off value expression	Expression	*P*-value	HR	Number of patients
CCL1	9	1–240	6.7e-6	0.78 (0.7–0.87)	3951
CCL2	680	4–18188	0.8939	1.01 (0.9–1.12)	3951
CCL3	515	9–13339	5.4e-6	0.78 (0.7–0.87)	3951
CCL4	469	4–10285	4.4e-10	0.71 (0.63–0.79)	3951
CCL5	655	25–22269	0.0584	0.9 (0.8–1.0)	3951
CCL7	46	1–1874	0.2592	1.06 (0.95–1.18)	2519
CCL8	375	10–8204	0.0025	1.18 (1.06–1.32)	3951
CCL11	129	1–1956	1.5e-5	0.79 (0.71–0.88)	3951
CCL13	121	2–7100	0.0022	0.84 (0.76–0.94)	3951
CCL14	464	3–13810	8.1e-10	0.71 (0.64–0.79)	3951
CCL15	464	3–13810	8.1e-10	0.71 (0.64–0.79)	3951
CCL16	39	1–2126	0.0001	0.81 (0.73–0.9)	3951
CCL17	32	2–663	0.5154	0.96 (0.86–1.08)	3951
CCL18	273	3–23425	0.027	1.13 (1.01–1.26)	3951
CCL19	706	3–18958	1.3e-7	0.75 (0.67–0.83)	3951
CCL20	16	1–16387	0.6648	0.98 (0.88–1.09)	3951
CCL21	157	2–21466	0.0002	0.82 (0.73–0.91)	3951
CCL22	87	3–2842	0.0002	0.81 (0.73–0.91)	3951
CCL23	21	1–763	0.3072	0.94 (0.85–1.05)	3951
CCL24	24	2–419	0.1555	0.92 (0.83–1.03)	3951
CCL25	34	2–569	2.7e-6	0.77 (0.69–0.86)	3951
CCL26	33	1–969	0.1748	0.9 (0.77–1.05)	1764
CCL27	35	1–1494	0.0314	0.89 (0.8–0.99)	3951
CCL28	174	4–6879	0.1106	0.88 (0.75–1.03)	1764

Event: RFS. Abbreviation: HR, hazard ratio.

**Table 3:** Prognostic value of CC chemokines expression in BC (Kaplan–Meier plotter)

Chemokine	Cut-off value expression	Expression	*P*-value	HR	Number of patients
CCL1	10	1–240	0.977	1 (0.8–1.24)	1402
CCL2	748	9–18188	0.2119	0.87 (0.71–1.08)	1402
CCL3	521	9–13339	0.0186	0.77 (0.62–0.96)	1402
CCL4	505	21–10285	0.027	0.72 (0.58–0.89)	1402
CCL5	768	55–22269	0.2046	0.87 (0.7–1.08)	1402
CCL7	48	1–1374	0.3076	1.12 (0.9–1.38)	1402
CCL8	420	10–8024	0.4704	1.08 (0.87–1.34)	1402
CCL11	137	3–1956	0.4449	0.92 (0.74–1.14)	1402
CCL13	130	4–4102	0.4672	0.92 (0.75–1.14)	1402
CCL14	465	3–6969	0.0008	0.69 (0.56–0.86)	1402
CCL15	465	3–6969	0.0008	0.69 (0.56–0.86)	1402
CCL16	37	1–2126	0.8236	1.02 (0.83–1.27)	1402
CCL17	27	2–663	0.022	1.28 (1.04–1.59)	1402
CCL18	293	5–23425	0.0156	1.3 (1.05–1.61)	1402
CCL19	848	14–15898	0.0015	0.71 (0.57–0.88)	1402
CCL20	17	1–16387	0.3747	1.11 (0.89–1.37)	1402
CCL21	159	2–21466	0.0031	0.72 (0.58–0.9)	1402
CCL22	113	3–2618	0.0183	0.77 (0.62–0.96)	1402
CCL23	20	1–763	0.5297	1.07 (0.87–1.33)	1402
CCL24	23	2–419	0.007	1.34 (1.08–1.66)	1402
CCL25	37	2–569	0.4578	1.08 (0.88–1.34)	1402
CCL26	38	1–969	0.6008	1.09 (0.8–1.49)	626
CCL27	30	1–704	0.1243	1.18 (0.95–1.46)	1402
CCL28	164	4–2786	0.5701	1.11 (0.81–1.52)	626

Event: OS. Abbreviation: HR, hazard ratio.

### The correlation between aberrantly expressed CC chemokines and clinicopathological parameter of BC patients

**Figure 5:**
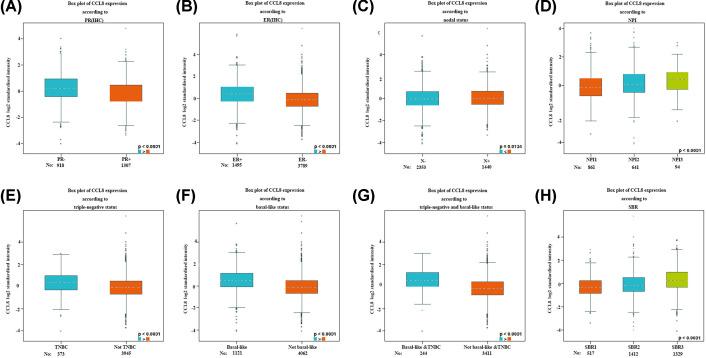
The correlation between CCL8 and clinicopathological parameter of BC patients High CCL8 expression was significantly correlated with negative PR, negative ER, positive nodal status, triple-negative BC subtype, basal-like BC subtype, triple-negative and basal-like BC subtype and high grades.

The correlation between CCL8 and clinicopathological parameters of BC patients is shown in Figure 5. BC patients with high CCL8 are more likely to have a negative PR (Figure 5A, *P*<0.0001), positive ER (Figure 5B, *P*<0.0001) and lymph node metastasis (Figure 5C, *P*=0.0134). Significant up-regulation of CCL8 was revealed in patients with triple-negative BC (Figure 5E, *P*<0.0001), basal-like BC (Figure 5F, *P*<0.0001) and triple-negative and basal-like BC (Figure 5G, *P*<0.0001). The Nottingham Prognostic Index (NPI, Figure 5D) and the Scarff, Bloom and Richardson grade (SBR, Figure 5H) are favorable prognostic models for BC. According to NPI and SBR criteria, significant up-regulation of CCL8 was revealed in BC patients with poorly differentiated tumors (grades II and III) compared with those with well-differentiated tumors (grade I) (*P*<0.0001). These results demonstrate that high CCL8 expression is associated with bad prognostic clinicopathologic features.

**Figure 6:**
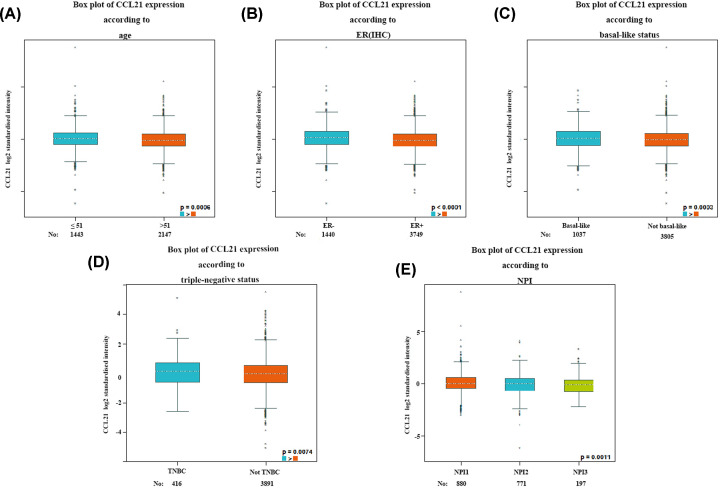
The correlation between CCL21 and clinicopathological parameter of BC patients High levels of CCL21 were associated with negative PR, triple-negative subtype and basal-like subtype and low tumor grade.

The correlation between CCL21 and clinicopathological parameters of BC patients are shown in Figure 6. For age and IHC criterion, BC patients less than 51 years old (Figure 6A, *P*=0.0006) or negative PR (Figure 6B, *P*<0.0001) tended to express high levels of CCL21. Moreover, basal-like BC and triple-negative BC patients had significant up-regulation of CCL21 (Figure 6C,D, *P*<0.0001). According to NPI criterion, BC patients with poorly differentiated tumors (grades II and III) had significantly lower expression of CCL21 compared with those with well-differentiated tumors (grade I) (Figure 6E, *P*=0.0011).

### CCL8/21 genetic alteration, neighbor gene network and interaction analyses in BC patients

**Figure 7:**
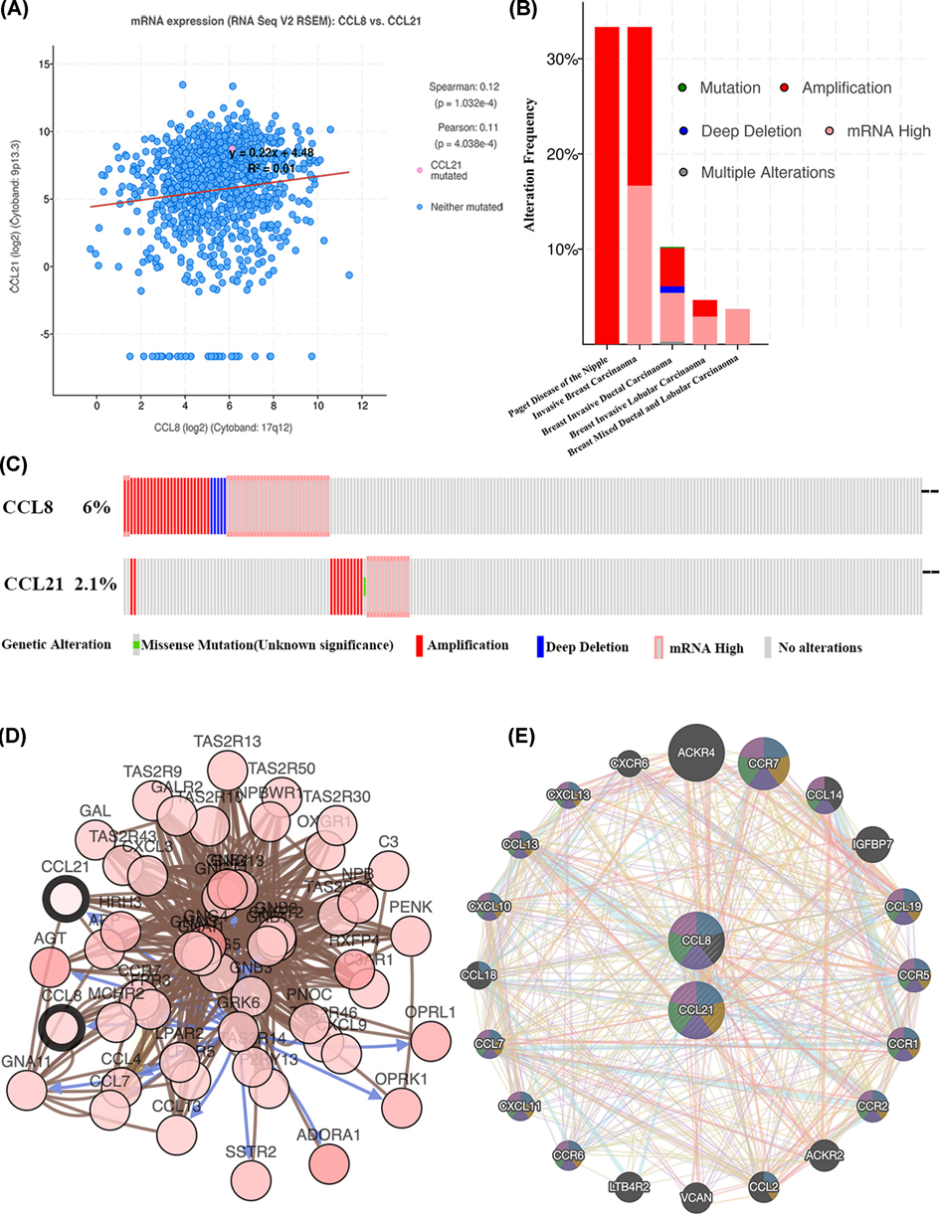
Genetic alteration, neighbor gene network and interaction analyses of CCL8/21 in BC patients (**A**) The relationship of CCL8 and CCL21 in mRNA expression. (**B**) Summary of alterations in CCL8/21 in BC types. (**C**) OncoPrint visual summary of alteration on a query of CCL8/21. (**D**) Gene–gene interaction network of CCL8/21 and 50 most frequently altered neighboring genes. (**E**) Protein–protein interaction network of CCL8/21 in the GeneMANIA dataset.

Due to the significant clinical value of CCL8/21, we embarked on a comprehensive exploration of these chemokines’ molecular characteristics. cBioPortal was utilized to analyze the molecular characteristics of CCL8/21 in BC. In our study, a total of 1085 BC patients were included in TCGA and provisional datasets. Data from TCGA revealed that CCL8 and CCL21 were co-expressed with a Pearson correlation coefficient of 0.11 (Figure 7A, *P*=4.038E-4). Among the cancer type study, CCL8/21 was altered in 1/3 (33%) patients with Paget disease of the nipple, 2/6 (30%) with invasive breast carcinoma, 74/723 (10.24%) with breast invasive ductal carcinoma, 8/192 (4.65%) with breast invasive lobular carcinoma, and 1/27 (3.7%) with breast invasive mixed ductal lobular carcinoma (Figure 7B).

Next, we analyzed the genetic alteration of CCL8 and CCL21 in BC. Results indicated that CCL8 was altered in 65 (6%) and CCL21 was altered in 29 (2.7%) of 1085 TCGA BC patients (Figure 7C). Additionally, we constructed the network for CCL8/21 and the 50 most frequently altered neighbor genes, which revealed that *ADORA1, AGT, APP, C3AR1, CCL13, CCL4, CCL7, CCR7, CXCL3, CXCL9, FPR3, GAL, GALR2, GNA11, GNAI1, GNAI3, GNB1, GNB2, GNB3, GNB4, GNG13, GNG4, GNG5, GNGT1, GNGT2, GRK6, HRH3, LPAR2, LPAR5, MCHR2, NPB, NPBWR1, OPRK1, OPRL1, OXGR1, P2RY13, PENK, PNOC, RXFP4, SSTR2, TAS2R10, TAS2R13, TAS2R14, TAS2R30, TAS2R31, TAS2R43, TAS2R46, TAS2R50, TAS2R9* were closely associated with CCL8/21 genetic alterations (Figure 7D). Moreover, PPI network analysis of CCL8/21 at the gene level performed by GeneMANIA revealed that the function of CCL8 and CCL21 was mainly associated with cell chemotaxis, leukocyte chemotaxis, cellular calcium ion homeostasis and chemokine-mediated signaling pathway (Figure 7E).

### Functional enrichment analysis of CCL8/21 in BC patients

**Figure 8:**
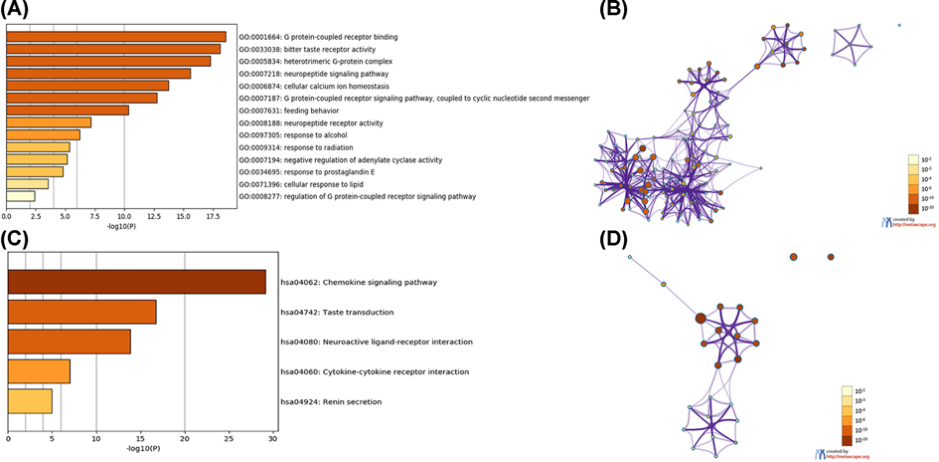
The enrichment analysis of CCL8/21 and 50 most frequently altered neighboring genes in BC (Metascape) (**A**) Heatmap of GO enriched terms colored by *P*-values. (**B**) Network of GO enriched terms colored by *P*-value, where terms containing more genes tend to have a more significant *P*-value. (**C**) Heatmap of KEGG enriched terms colored by *P*-values. (**D**) Network of KEGG enriched terms colored by *P*-value, where terms containing more genes tend to have a more significant p-value.

Due to the significant clinical utility of CCL8/21, we embarked on a comprehensive exploration of their function. Functional enrichment analysis of CCL8/21 and the neighboring genes were performed in GO and KEGG via Metascape and David 6.8. The results of 14 GO enrichment items in Metascape are shown in Figure 8A,B and Supplementary Table S1. Overall, ten pathways of biological process, one of cellular component and three of molecular function were obtained. CCL8/21 and their neighboring genes were mainly enriched in G protein-coupled receptor and receptor activity, which is associated with tumorigenesis and progression of BC. Figure 8C,D and Supplementary Table S2 show the top five KEGG pathways for CCL8/21 and their neighboring genes. Among the five KEGG pathways, chemokine signaling pathway and cytokine–cytokine receptor interaction were involved in BC tumorigenesis and pathogenesis.

**Figure 9:**
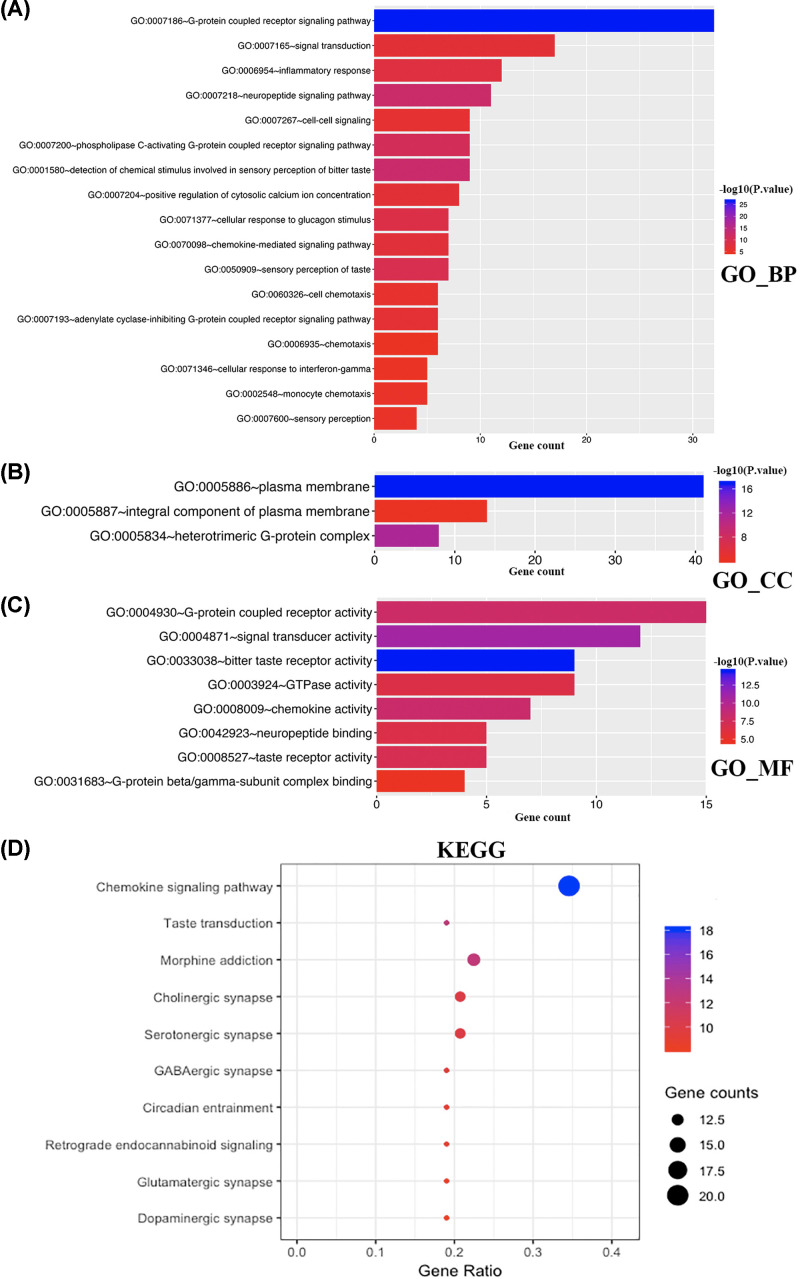
The enrichment analysis of CCL8/21 and 50 most frequently altered neighboring genes in BC (DAVID 6.8) (**A**) Heatmap of GO enrichment in cellular component terms, biological process terms and molecular function terms. (**B**) Bubble map of KEGG enriched terms.

Results from functional enrichment analysis of DAVID 6.8 were shown in Figure 9. In total, 17 pathways involved in biological process (Figure 9A), 3 pathways involved in cellular component (Figure 9B) and 8 pathways involved in molecular function (Figure 9C) were obtained from GO enrichment analysis. CCL8/21 and their neighboring genes were mainly enriched in G protein-coupled receptor, inflammatory response, signal transducer and chemokine activity, which were consistent with results of Metascape. Additionally, G protein-coupled receptor, inflammatory response, signal transducer and chemokine activity are associated with BC tumorigenesis and pathogenesis. Among the ten KEGG pathways (Figure 9D), chemokine signaling pathway, serotonergic synapse and circadian entrainment are associated with BC tumorigenesis and pathogenesis.

### CCL8/21 networks of kinase, miRNA or transcription factor targets in BC

To further explore the targets of CCL8/21 in BC, we analyzed the kinase, miRNA and transcription factor target networks of positively correlated gene sets generated by GSEA. Results of CCL21 are present in Table 4. FYN, LYN, LCK, CSNK2A1, and HCK are the top five most significant targets of the CCL8 kinase–target network. MIR-380-5P (GTCAACC) and MIR-155 (AGCATTA) were suggested as significant targets in the CCL8 miRNA–target network. In the transcription factor–target network, ETS_Q4, NFKB_Q6, COREBINDINGFACTOR_Q6, NFKAPPAB65_01, and NFKAPPAB_01 are the top five most significant targets. The results from CCL21 are present in Table 5. PLK1, FYN, CHEK2, PRKCA and WEE1 were the top five most significant targets in the CCL21 kinase–target network. The CCL21 miRNA–target network was related mainly to MIR-433 (ATCATGA), MIR-490 (CCAGGTT), MIR-346 (GGCAGAC), MIR-146A (AGTTCTC), MIR-146B, and MIR-453 (ACAACCT). In the transcription factor–target network, ETS_Q4, E2F_02, E2F1DP1_01, E2F1DP2_01 and E2F4DP2_01 are the top five most significant targets.

**Table 4:** The kinase, miRNA and transcription factor–target networks of CCL8 in BC (LinkedOmics)

Enriched category	Geneset	LeadingEdgeNum	*P*-value
Kinase Target	Kinase_FYN	7	0
	Kinase_LYN	5	0.008
	Kinase_LCK	4	0.014
	Kinase_CSNK2A1	18	0.017
	Kinase_LCK	1	0.018
miRNA Target	GTCAACC, MIR-380-5P	1	0
	AGCATTA, MIR-155	6	0.051
Transcription Factor Target	V$ETS_Q4	26	0
	V$NFKB_Q6	14	0
	V$COREBINDINGFACTOR_Q6	11	0
	V$NFKAPPAB65_01	16	00
	V$NFKAPPAB_01	17	0

**Table 5:** The kinase, miRNA and transcription factor–target networks of CCL21 in BC (LinkedOmics)

Enriched category	Geneset	LeadingEdgeNum	*P*-value
Kinase Target	Kinase_PLK1	17	0
	Kinase_FYN	11	0
	Kinase_CHEK2	4	0
	Kinase_PRKCA	18	0.004
	Kinase_WEE1	4	0.022
miRNA Target	ATCATGA, MIR-433	12	0
	CCAGGTT, MIR-490	4	0.013
	GGCAGAC, MIR-346	4	0.014
	AGTTCTC, MIR-146A, MIR-146B	3	0.030
	ACAACCT, MIR-453	3	0.031
Transcription Factor Target	V$ETS_Q4	20	0
	V$E2F_02	15	0
	V$E2F1DP1_01	15	0
	V$E2F1DP2_01	15	0
	V$E2F4DP2_01	15	0

### The correlation between CCL8/21 and immune cell infiltration

**Figure 10:**
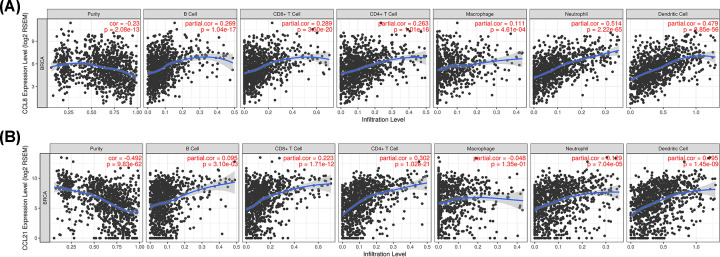
The correlation between CCL8/21 expression and immune cell infiltration (**A**)The correlation between CCL8 expression and immune cell infiltration. (**B**)The correlation between CCL21 expression and immune cell infiltration.

As the CC chemokines are involved in inflammatory responses and immune cell infiltration, and thus affect clinical outcomes in BC, we embarked on a comprehensive exploration of the correlation between CCL8/21 and immune cell infiltration using the TIMER database. As expected, there was a positive correlation between CCL8 expression and abundance of B cells (Cor = 0.269, *P*=1.04e-7), CD8^+^ T cells (Cor = 0.289, *P*=3.50e-20), CD4^+^ T cells (Cor = 0.263, *P*=1.01e-16), macrophages (Cor = 0.111, *P*=4.61e-4), neutrophils (Cor = 0.514, *P*=2.22e-65) and dendritic cells (Cor = 0.479, *P*=8.85e-56) (Figure 10A). Furthermore, a negative correlation was obtained between CCL8 expression and BC tumor purity (Cor = −0.23, *P*=2.08e-13) (Figure 10A). In the CCL21 category, we found a positive correlation between CCL21 expression and the abundance of B cells (Cor = 0.095, *P*=3.01e-3), CD8^+^ T cells (Cor = 0.223, *P*=1.71e-12), CD4^+^ T cells (Cor = 0.302, *P*=1.02e-21), neutrophils (Cor = 0.129, *P*=7.04e-5) and dendritic cells (Cor = 0.195, *P*=1.45e-9) (Figure 10B). We observed a negative correlation between CCL21 expression and BC tumor purity (Cor = −492, *P*=9.83e-62) (Figure 10B). We also explored the clinical relevance of CCL8/21 and immune cell infiltration using a cox proportional hazard model, which corrects for potential confounding factors. After correcting for gender, race and tumor purity, we identified that B cells, CD8^+^ T cell, CD4^+^ T cell, neutrophils, dendritic cells, CCL21, age (*P*=0), tumor stage (*P*=0), macrophage infiltration (*P*=0.035) and CCL8 (*P*=0.033) were associated with clinical outcomes of BC (Supplementary Table S1).

## Discussion

Chemokines are a family of small proteins that are produced by several cell types including tumor cells, and exert immunologic functions by binding to G-protein coupled receptors (GPCRs) [31]. Cumulative evidence demonstrates that aberrant expression of chemokines is involved in the tumorigenesis and progression of several cancers, including BC [32,33]. Additionally, chemokines secreted by the tumor microenvironment are the driving force behind trafficking of immune cells into the primary tumor. Therefore, cross-talk between tumor cells and infiltrating immune cells plays a significant role in tumor invasion and metastasis [34]. As the largest family of chemokines, CC chemokines have been reported to play a significant role in BC [15]. However, the expression and the exact function of all CC chemokines members in BC are far from clear. In this current study, we systematically explored the expression patterns, prognostic values and potential functions of differentially expressed CC chemokines in BC.

CCL8, also known as MCF2, acts as an agonist of the CC chemokine receptor type 2 (CCR2) and CCR5, exerting significant function in the process of leukocyte chemotaxis, cancer tumorigenesis and progression, and responds to changes in the pathogenic environment [35,36]. CCL8 is involved in the regulation of many cellular processes, such as proliferation, apoptosis, and differentiation [37–40]. CCL8 has shown tremendous potential in the migration of inflammatory cells and anti-tumoral effects [29]. Furthermore, Hiwatashi et al*.* demonstrated that CCL8 induces tumor-promoting inflammatory reactions and anti-tumor effects by attracting tumor-associated macrophages [36]. Previous study revealed that CCL8 can induce epithelial–mesenchymal transition (EMT) in esophageal squamous cell carcinoma cells, thus facilitating tumor cell migration and invasion [39]. In melanoma, cell-to-cell communication in tumor cells impacts CCL8 expression patterns, thus building a microenvironment that facilitates tumor migration and metastasis [41]. Moreover, CCL8 not only facilitates tumor invasion to adjacent stroma in BC but also intravasation and extravasation, leading to the establishment of secondary growth [42]. In our study, CCL8 expression was significantly up-regulated in BC tissues compared with normal tissues. Furthermore, high CCL8 expression was significantly correlated with negative PR, negative ER, positive nodal status, triple-negative BC subtype, basal-like BC subtype, triple-negative and basal-like BC subtype and high grades. In addition, high CCL8 expression was significantly related to worse prognosis among patients with BC.

CCL21, also known as 6Ckine/SLC, acts as an agonist of CCR7 and mediates DC and T cell homing to human tissues [36]. Previous studies have revealed that CCL21 may facilitate infiltration of immune cells into the tumor area, leading to the production of an anti-tumor cell immune response and suppression of tumor growth [43,44]. Besides, in combination with tumor-infiltrating CD8^+^ T cells, CCL21-expressing DCs can mediate tumor eradication [45]. Another study demonstrated that CCL21 secreted by tumor cells can establish an immune tolerant tumor microenvironment, thus promoting tumor progression [46]. CCL21 functions as a pivotal regulator of cell proliferation, invasion, apoptosis and tumor metastasis [47–50]. In lung cancer, CCL21/CCR7 triggers tumor cell migration and invasion via the EMT and ERK1/2 signaling pathway, thus providing a potential target for lung cancer treatment [51]. CCL21/CCR7 enhanced bladder cancer cell proliferation and facilitated migration and invasion by increasing levels of MMP-2 and MMP-9 [52]. The prognostic value of CCL21 in cancer patients is significant varies according to the tumor type. Previous studies have revealed that up-regulation of CCL21 is a favorable prognostic factor in pancreatic cancer, renal cell carcinoma, colorectal cancer and BC, and represents a negative prognostic factor in bladder cancer and gastric cancer [49,53,54]. In our study, CCL21 was down-regulated in BC, and high levels of CCL21 was associated with negative PR, triple-negative subtype and basal-like subtype and low tumor grade. Additionally, low CCL21 expression was significantly related to worse prognosis among patients with BC.

Increasingly more findings have demonstrated that the cross-talk of CC chemokines, including CCL8 and CCL21, in BC is significantly associated with carcinogenesis, tumor metastasis and chemoresistance. The studies of these CC chemokines may provide potential drug treatment targets. In our study, we selected CCL8 and CCL21 as our research target due to their abnormal expression and prognostic value in BC. A positively co-expressed relationship was seen in CCL8 and CCL21. Furthermore, the PPI networks revealed that the functions of CCL8 and CCL21 was significantly enriched in cellular and leukocyte chemotaxis. Previous studies have demonstrated the significant role of cellular and leukocyte chemotaxis in the tumorigenesis and prognosis of BC. Pang et al. [55]. suggested that TGF-β1-induced EMT can activate tumor cell migration by modeling CCR7/CCL21-mediated chemotaxis in BC [55]. High neutrophil to lymphocyte ratio was correlated to poor prognosis of BC patients [56]. Results of GO enrichment analysis and KEGG pathways revealed that CCL8 and CCL21 mainly function in chemokine signaling pathway, G protein-coupled receptor and inflammatory response, which are associated with BC carcinogenesis, tumor immune escape and chemoresistance. Brummer et al*.* demonstrated that chemokine signaling promoted BC survival and invasion by fibroblast-dependent mechanisms [57]. Accumulating evidence has demonstrated that high levels of tumor-infiltrating CD8^+^ T cells are associated with an improved prognosis and chemotherapy sensitivity in triple-negative BC [58]. These data demonstrate that CCL8 and CCL21 play a significant role in the tumorigenesis and progression of BC.

Another important finding of our study was that our result revealed methylation could down-regulate the level of CCL2/5/15/17/19/20/22/23/24/25/26/27 in BC. However, the expression levels of CCL2/3/4/5/7/8/11/17/19/20/22 genes have been shown as some up-regulated chemokines in BC tissues. These may indicate a negative correlation between DNA methylation and the expression of CCL2/5/17/19/20/22. These were consistent with the fact that DNA methylation plays an important role in the development of various cancers mainly through the regulation on gene expression [59]. Further study should be performed to clarify the role of DNA methylation and CCL2/5/17/19/20/22 in the generation and development of BC. Drug resistance revealed that low expression of CCL3/4/23 are associated with drug resistance. Actually, previous study has suggested that a pathway involving EBNA2/Btk/NF-κB/CCL3/CCL4 plays a key role in doxorubicin resistance. Moreover, evaluation of the CCL3 and CCL4 levels may be helpful for selecting B-cell lymphoma patients likely to benefit from doxorubicin treatment in combination with the velcade or ibrutinib [60]. Methylation and transcriptome changes was correlated with drug resistance in many cancers, including ovarian cancer [61], gastric cancer [62], and BC [63]. However, limited evidences have demonstrated the correlation between the methylation of CCL3/CCL4/CCL23 chemokines and drug resistance in BC. And further study should be performed to verify this result.

Genomic instability and mutation are the basic characteristics of cancer cells, while kinases and their related signaling pathways contribute to the stabilization and repair of genomic DNA [64,65]. In our study, we identified FYN, LYN, LCK, CSNK2A1, HCK, PLK1, CHEK2, PRKCA and WEE1 as significant kinases involved in the CCL8/21-associated kinase network in BC. These kinases regulate genomic stability, RNA transcription, mitosis and cell cycle [66–69]. Our study also identified several miRNAs (MIR-380-5P, MIR-155, MIR-433, MIR-490, MIR-346, MIR-146A, MIR-146B, and MIR-453) that were strongly associated with CCL8/21. miRNAs, a category of non-coding RNAs, are able to regulate gene expression at a post-transcriptional level, which affects human carcinogenesis [70]. Among the miRNAs, MIR-155, MIR-433, MIR-490, MIR-346 and MIR-146A were found to be involved in regulating various functions of BC cells including migration, invasion, proliferation and apoptosis [71–73]. Moreover, MIR-146A acts as prognostic biomarker in BC [74,75]. Transcription factors play a significant role in cell cycle disorder, leading to aberrant differentiation, proliferation and apoptosis of tumor cells [76,77]. In current study, ETS and E2F gene family transcription factors were found to be associated with CCL8/21 in BC. The ETS transcription factors family consists of 28 numbers and many of them have been implicated in the development and progression of BC [78,79]. Aberrant E2F1 expression is significantly involved in the tumorigenesis and progression of BC and elevated E2F1 expression is associated with poor prognosis in BC patients [80,81].

Previous studies have revealed that CC chemokines are involved in inflammatory responses and immune cell infiltration in BC, which affects patients’ clinical outcomes [82,83]. Certain studies have described the correlation between immune cell infiltration and prognosis of BC. High levels of CD4^+^ T cells have a negative prognostic role in the outcomes of BC patients [84]. Welm et al*.* suggested that the macrophage-stimulating protein pathway can promote tumor metastasis and is associated with a poor clinical outcome in BC [85]. Another study revealed that plasmacytoid dendritic cells predict poor prognosis in BC [86]. In this current study, a positive correlation was obtained between CCL8/21 expression and the abundance of specific immune cells including B cells, CD8^+^ T cells, CD4^+^ T cells, macrophages, neutrophils and dendritic cells. Interestingly, high neutrophils infiltration could promote BC metastasis [87]. Moreover, high neutrophils infiltration in BC was associated with poor prognosis [88]. In our study, a cox proportional hazard model suggested that CCL8 was associated with clinical outcomes BC. These suggested that CCL8 and neutrophils played a synergistic role in the occurrence and development of BC, thus affecting the prognosis of patients. And our results may provide additional data regarding the correlation among CC chemokine expression, immune cell infiltration and clinical outcome of BC patients.

There are some limitations in our study. First, transcriptome sequencing is only able to detect static mutations and thus cannot directly provide information on protein activity or expression level. Moreover, the prognostic value of CCL8/21 in BC was not verified by further experiments. Another limitation is that our study ignored the fact that the molecular signatures of BC subtypes are very different.

## Conclusion

In short, CCL8 and CCL21 were found to be aberrantly expressed in BC and are associated with patient clinical outcomes. Functional analysis demonstrated that CCL8 and CCL21 are involved in carcinogenesis, tumor immune escape mechanisms and chemoresistance in BC by several cancer-related kinases, miRNAs and transcription factors (ETS and E2F gene family). Significant correlation was found between CCL8/21 expression and immune cell infiltration. Our results lay a foundation for further study of functions of CCL8/21 in BC.

## Supplementary Material

Supplementary Figures S1-S6 and Tables S1-S6

## Data Availability

All data generated or analyzed during the present study are included in this published article.

## Abbreviations

BC: breast cancer
bcGenExMiner: BC Gene-Expression Miner
CCL: Chemokine (C-C motif) ligand
CCR: CC chemokine receptor
CXC: Chemokine (C-X-C motif) ligand
EMT: epithelial–mesenchymal transition
ER: estrogen
ETS: E-twenty-six
E2F1: E2F Transcription Factor 1
FDR: false discovery rate
GEPIA: Gene Expression Profiling Interactive Analysis
GO: Gene Ontology
GSEA: Gene Set Enrichment Analysis
IHC: Immunohistochemistry
KEGG: Kyoto Encyclopedia of Genes and Genomes
NPI: Nottingham Prognostic Index
TCGA: The Cancer Genome Atlas
OS: overall survival
PPI: Protein-Protein Interaction
PR: progesterone
RFS: relapse-free survival
SBR: Scarff, Bloom and Richardson grade
