# Supplementary material for: Bioinformatics identification of CCL8/21 as potential prognostic biomarkers in breast cancer microenvironment
Source: Biosci Rep. 2020 Nov 24;40(11):BSR20202042. doi: 10.1042/BSR20202042 (PMC7687043; doi:10.1042/BSR20202042)
Supplement: Supplementary Figures S1-S6 and Tables S1-S6 [file BSR-2020-2042_supp.pdf]

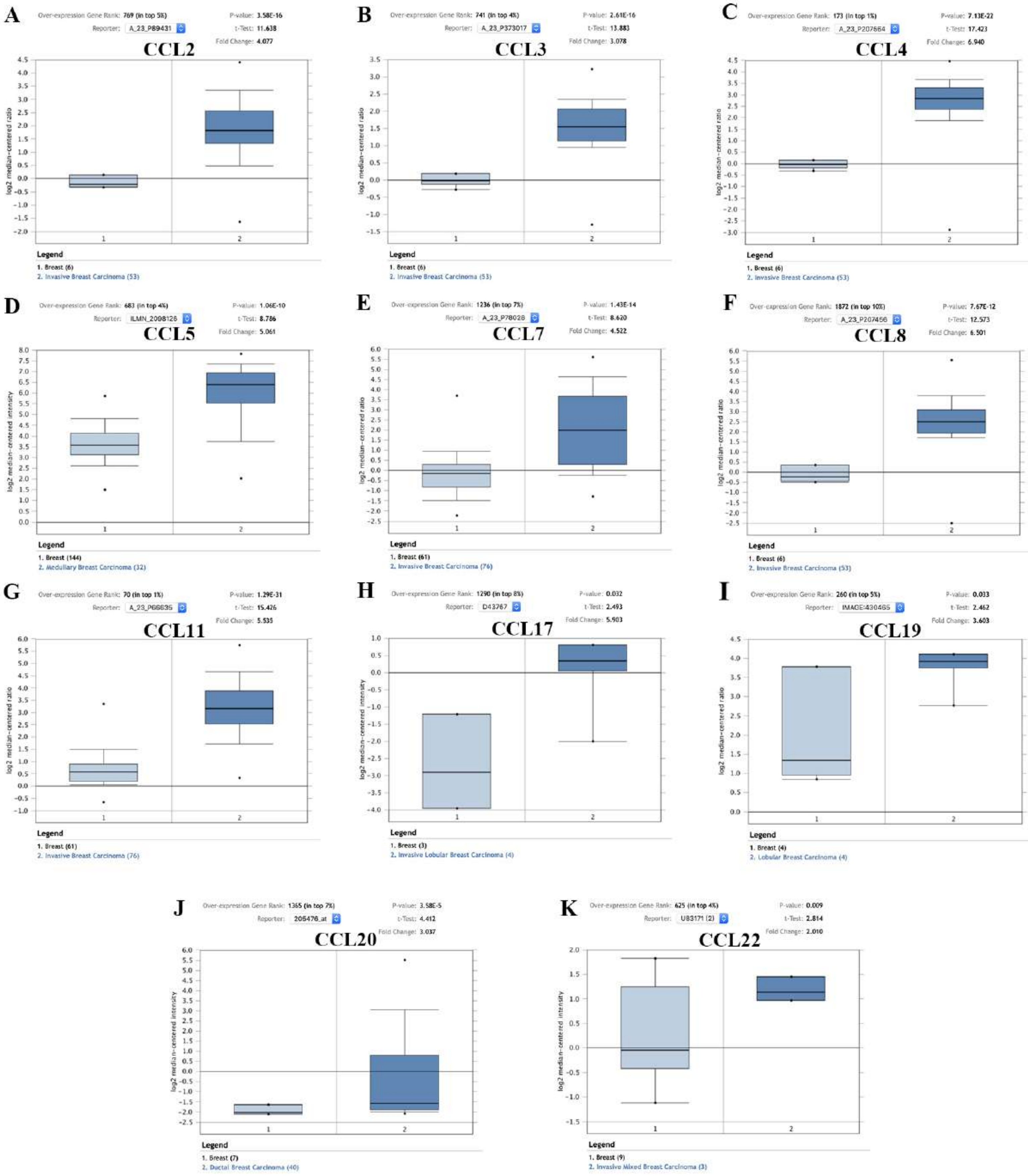

A
Over-expression Gene Rank: 769 (in top 5%)
Reporter: A_23_P89431
P-value: 3.58E-16
t-Test: 11.638
Fold Change: 4.077
CCL2
log2 median-centered ratio
Legend
1. Breast (6)
2. Invasive Breast Carcinoma (53)
B
Over-expression Gene Rank: 741 (in top 4%)
Reporter: A_23_P373017
P-value: 2.61E-16
t-Test: 13.883
Fold Change: 3.078
CCL3
log2 median-centered ratio
Legend
1. Breast (6)
2. Invasive Breast Carcinoma (53)
C
Over-expression Gene Rank: 173 (in top 1%)
Reporter: A_23_P207564
P-value: 7.13E-22
t-Test: 17.423
Fold Change: 6.940
CCL4
log2 median-centered ratio
Legend
1. Breast (6)
2. Invasive Breast Carcinoma (53)
D
Over-expression Gene Rank: 683 (in top 4%)
Reporter: ILMN_2098126
P-value: 1.06E-10
t-Test: 8.786
Fold Change: 5.061
CCL5
log2 median-centered intensity
Legend
1. Breast (144)
2. Medullary Breast Carcinoma (32)
E
Over-expression Gene Rank: 1236 (in top 7%)
Reporter: A_23_P78028
P-value: 1.43E-14
t-Test: 8.620
Fold Change: 4.522
CCL7
log2 median-centered ratio
Legend
1. Breast (61)
2. Invasive Breast Carcinoma (76)
F
Over-expression Gene Rank: 1872 (in top 10%)
Reporter: A_23_P207456
P-value: 7.67E-12
t-Test: 12.573
Fold Change: 6.501
CCL8
log2 median-centered ratio
Legend
1. Breast (6)
2. Invasive Breast Carcinoma (53)
G
Over-expression Gene Rank: 70 (in top 1%)
Reporter: A_23_P66635
P-value: 1.29E-31
t-Test: 15.426
Fold Change: 5.535
CCL11
log2 median-centered ratio
Legend
1. Breast (61)
2. Invasive Breast Carcinoma (76)
H
Over-expression Gene Rank: 1290 (in top 8%)
Reporter: D43767
P-value: 0.032
t-Test: 2.493
Fold Change: 5.903
CCL17
log2 median-centered intensity
Legend
1. Breast (3)
2. Invasive Lobular Breast Carcinoma (4)
I
Over-expression Gene Rank: 260 (in top 5%)
Reporter: IMAGE:430465
P-value: 0.033
t-Test: 2.462
Fold Change: 3.603
CCL19
log2 median-centered ratio
Legend
1. Breast (4)
2. Lobular Breast Carcinoma (4)
J
Over-expression Gene Rank: 1365 (in top 7%)
Reporter: 205476_at
P-value: 3.58E-5
t-Test: 4.412
Fold Change: 3.037
CCL20
log2 median-centered intensity
Legend
1. Breast (7)
2. Ductal Breast Carcinoma (40)
K
Over-expression Gene Rank: 625 (in top 4%)
Reporter: U83171 (2)
P-value: 0.009
t-Test: 2.814
Fold Change: 2.010
CCL22
log2 median-centered intensity
Legend
1. Breast (9)
2. Invasive Mixed Breast Carcinoma (3)

SupplementaryFig1. The level of CC chemokines in breast cancer patients (ONCOMINE). CCL2(A), CCL3(B), CCL4 (C), CCL5(D), CCL7(E), CCL8(F), CCL11(G), CCL17(H), CCL19(I), CCL20(J), CCL22(K) were significant upregulated in breast cancer tissues compared with normal tissues; *P<0.05.

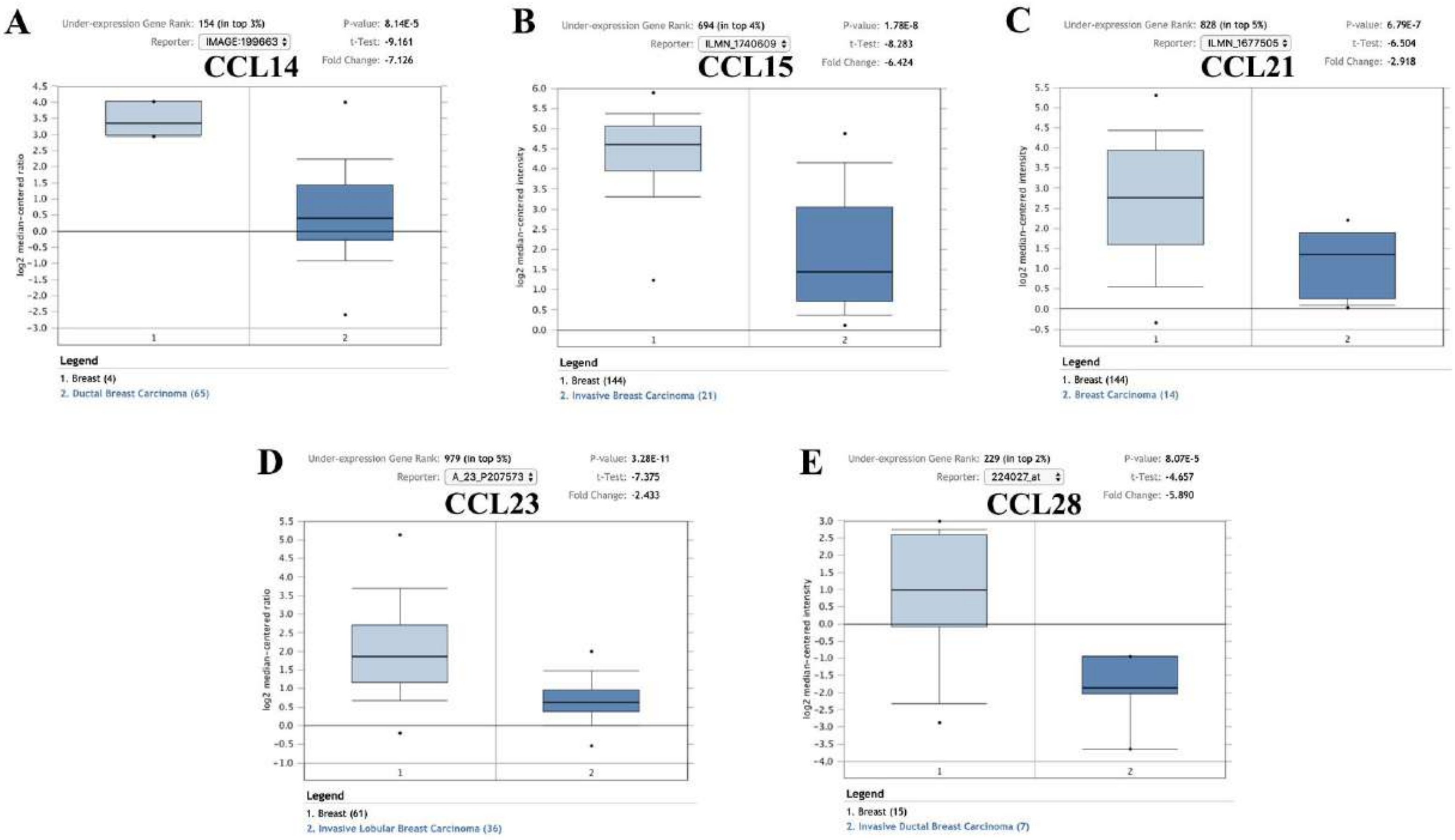
A
Under-expression Gene Rank: 154 (in top 3%)
Reporter: IMAGE:199663
P-value: 8.14E-5
t-Test: -9.161
Fold Change: -7.126
CCL14
log2 median-centered ratio
Legend
1. Breast (4)
2. Ductal Breast Carcinoma (65)
B
Under-expression Gene Rank: 694 (in top 4%)
Reporter: ILMN_1740609
P-value: 1.78E-8
t-Test: -8.283
Fold Change: -6.424
CCL15
log2 median-centered intensity
Legend
1. Breast (144)
2. Invasive Breast Carcinoma (21)
C
Under-expression Gene Rank: 828 (in top 5%)
Reporter: ILMN_1677505
P-value: 6.79E-7
t-Test: -6.504
Fold Change: -2.918
CCL21
log2 median-centered intensity
Legend
1. Breast (144)
2. Breast Carcinoma (14)
D
Under-expression Gene Rank: 979 (in top 5%)
Reporter: A_23_P207573
P-value: 3.28E-11
t-Test: -7.375
Fold Change: -2.433
CCL23
log2 median-centered ratio
Legend
1. Breast (61)
2. Invasive Lobular Breast Carcinoma (36)
E
Under-expression Gene Rank: 229 (in top 2%)
Reporter: 224027_at
P-value: 8.07E-5
t-Test: -4.657
Fold Change: -5.890
CCL28
log2 median-centered intensity
Legend
1. Breast (15)
2. Invasive Ductal Breast Carcinoma (7)

SupplementaryFig 2. The level of CC chemokines in breast cancer patients. CCL14(A), CCL15(B), CCL21(C), CCL23(D), CCL28(E) were significant downregulated in breast cancer tissues compared with normal tissues; *P<0.05.

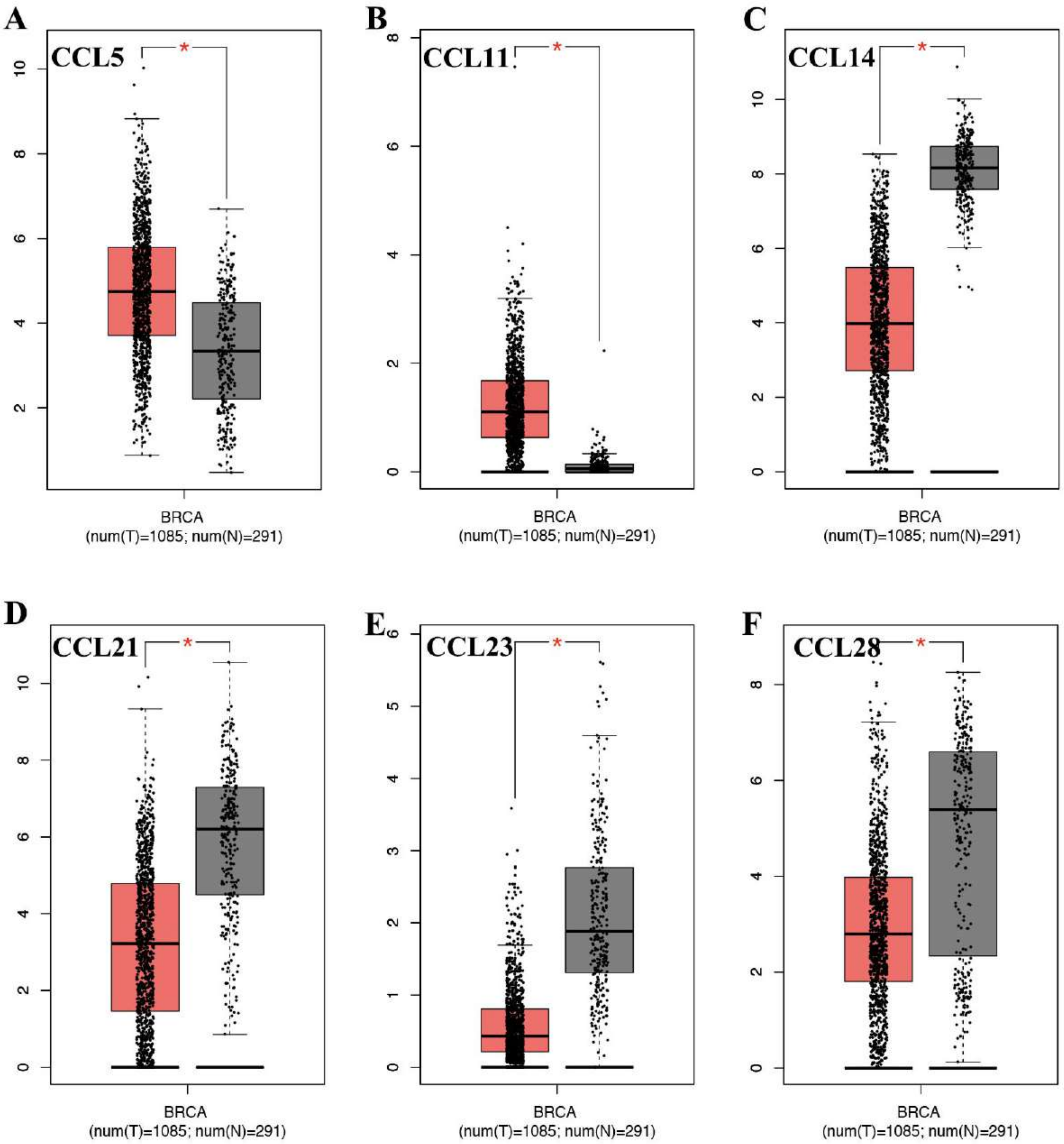
A
CCL5
B
CCL11
C
CCL14
D
CCL21
E
CCL23
F
CCL28
BRCA
(num(T)=1085; num(N)=291)

Supplementary Fig 3. The level of CC chemokines in breast cancer patients with the threshold of P-value<0.05 (GEPIA). The figure shows the significant aberrant expression of CCL5(A), CCL11(B), CCL14(C), CCL21(D), CCL23(E), CCL28(F). Num, number; T, tumor tissues; N, normal tissues; *P<0.05.

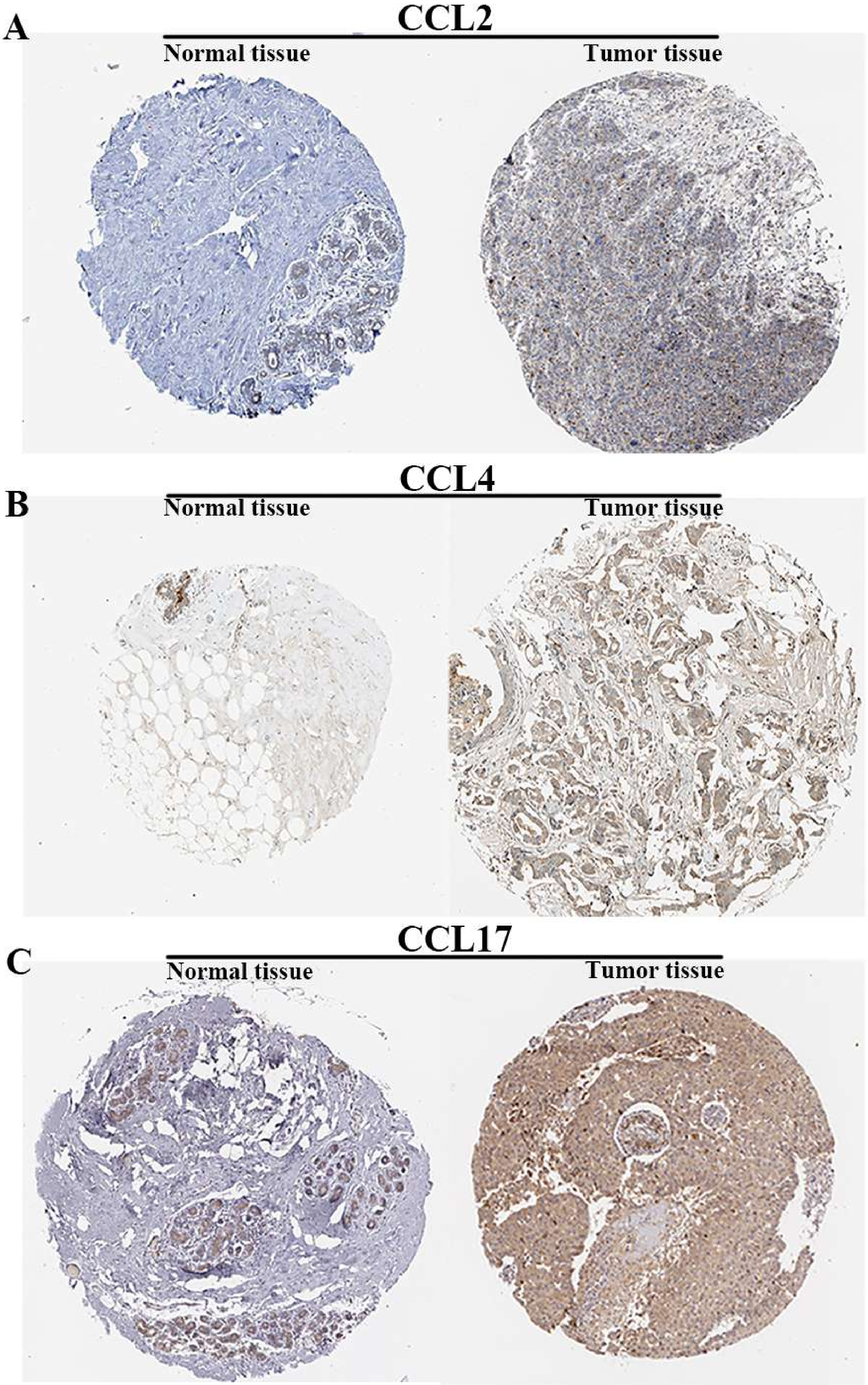

A
CCL2
Normal tissue
Tumor tissue
B
CCL4
Normal tissue
Tumor tissue
C
CCL17
Normal tissue
Tumor tissue

Supplementary Fig 4. The level of CC chemokines in breast cancer patients in protein level (The Human Protein Atlas). CCL2(A), CCL4(B), CCL17(C) were significant upregulated in breast cancer tissues compared with normal tissues.

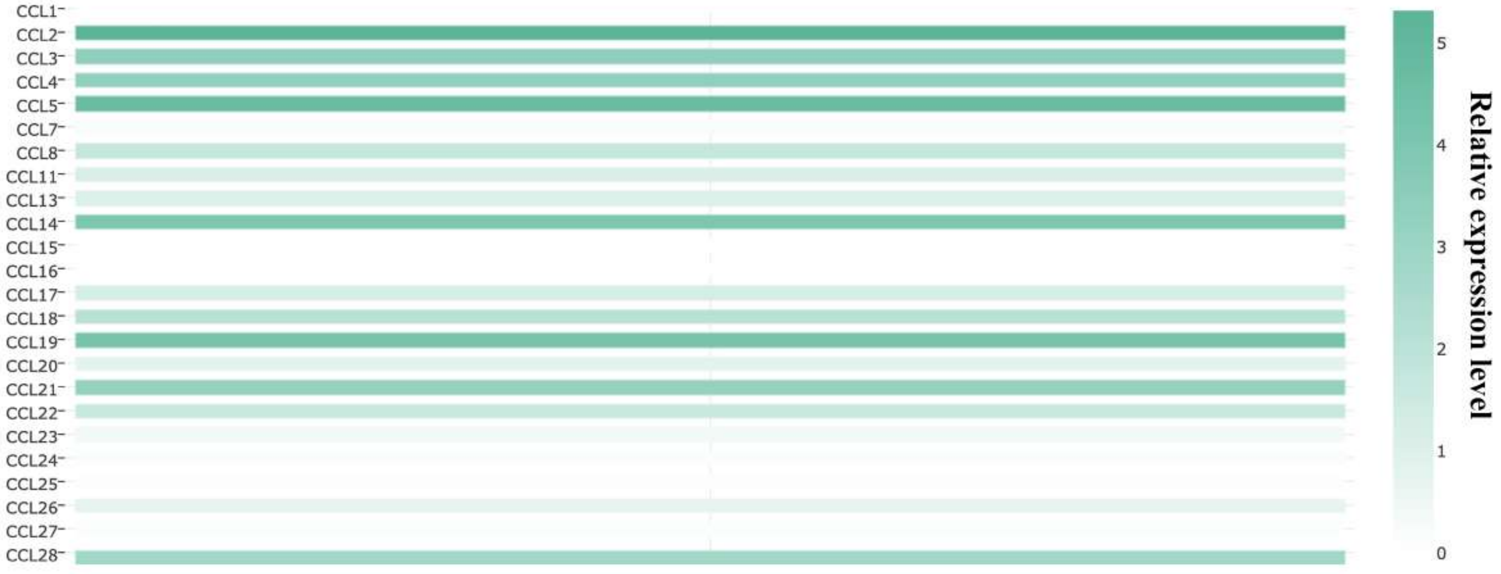
CCL1
CCL2
CCL3
CCL4
CCL5
CCL7
CCL8
CCL11
CCL13
CCL14
CCL15
CCL16
CCL17
CCL18
CCL19
CCL20
CCL21
CCL22
CCL23
CCL24
CCL25
CCL26
CCL27
CCL28
Relative expression level
5
4
3
2
1
0

Supplementary Fig 5. The relative expression level of aberrant expression of CC chemokines in breast cancer tissues.The darker the color of green, the higher the level of CC chemokines expression.

High expression of chemokines predict good prognosis

High expression of chemokines predict poor prognosis

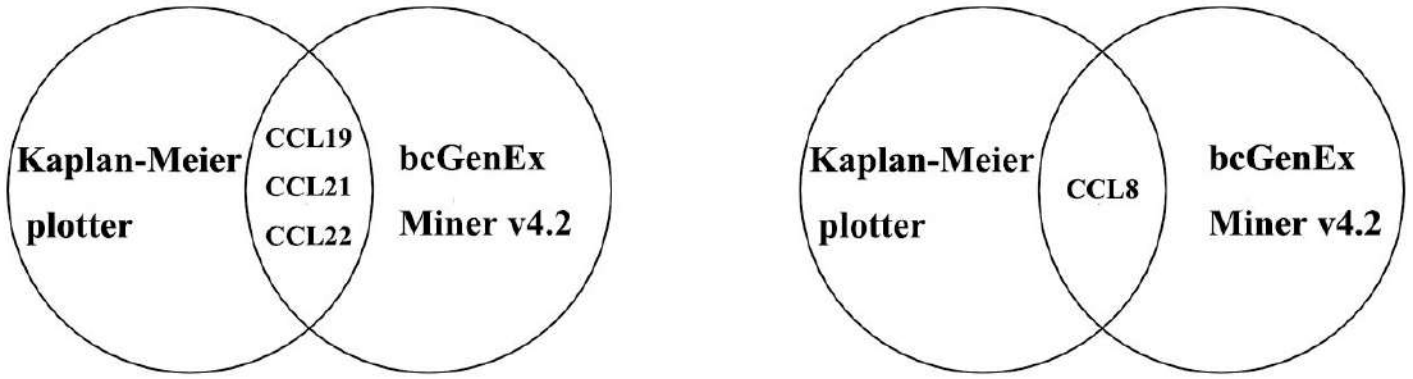

Supplementary Fig 6. Venn diagram demonstrated that CCL8/19/21/22 are associated with clinical out come in breast cancer.

Table 1. Prognostic value of CC chemokines expression in breast cancer (bc-GenExMiner v4.2).

Chemokine	Event	p-value	HR	95%CI
CCL1	MR	0.2212	0.92	0.81-1.05
CCL2	MR	0.2187	1.08	0.96-1.22
CCL3	MR	0.9834	1	0.82-1.22
CCL4	MR	0.1963	0.92	0.81-1.04
CCL5	MR	0.1908	0.92	0.82-1.04
CCL7	MR	0.1926	1.09	0..96-1.24
CCL8	MR	0.0018	1.23	1.08-1.39
CCL11	MR	0.8628	0.99	0.87-1.12
CCL13	MR	0.1432	1.00	0.97-1.25
CCL14	MR	0.8892	0.97	0.65-1.46
CCL15	MR	0.1885	1.29	0.88-1.89
CCL16	MR	0.0740	0.89	0.79-1.01
CCL17	MR	0.5476	1.04	0.92-1.18
CCL18	MR	0.3420	1.06	0.94-1.20
CCL19	MR	0.0015	0.82	0.72-0.93
CCL20	MR	0.2311	1.08	0.95-1.22
CCL21	MR	0.0006	0.81	0.71-0.91
CCL22	MR	0.0001	0.77	0.67-0.87
CCL23	MR	0.0056	0.83	0.73-0.95
CCL24	MR	0.6538	0.97	0.85-1.10
CCL25	MR	0.1051	1.11	0.98-1.26
CCL26	MR	0.4829	1.06	0.89-1.27
CCL27	MR	0.4840	0.96	0.84-1.09
CCL28	MR	0.8222	0.98	0.79-1.21

Event: metastatic recurrence. MR, metastatic recurrence; HR, Hazard radio

Table 2. Prognostic value of CC chemokines expression in breast cancer (Kaplan-Meier Plotter).

Chemokine	Cutoff value expression	Expression	P value	HR	No. Patients
CCL1	9	1-240	6.7e-6	0.78(0.7-0.87)	3951
CCL2	680	4-18188	0.8939	1.01(0.9-1.12)	3951
CCL3	515	9-13339	5.4e-6	0.78(0.7-0.87)	3951
CCL4	469	4-10285	4.4e-10	0.71(0.63-0.79)	3951
CCL5	655	25-22269	0.0584	0.9(0.8-1.0)	3951
CCL7	46	1-1874	0.2592	1.06(0.95-1.18)	2519
CCL8	375	10-8204	0.0025	1.18(1.06-1.32)	3951
CCL11	129	1-1956	1.5e-5	0.79(0.71-0.88)	3951
CCL13	121	2-7100	0.0022	0.84(0.76-0.94)	3951
CCL14	464	3-13810	8.1e-10	0.71(0.64-0.79)	3951
CCL15	464	3-13810	8.1e-10	0.71(0.64-0.79)	3951
CCL16	39	1-2126	0.0001	0.81(0.73-0.9)	3951
CCL17	32	2-663	0.5154	0.96(0.86-1.08)	3951
CCL18	273	3-23425	0.027	1.13(1.01-1.26)	3951
CCL19	706	3-18958	1.3e-7	0.75(0.67-0.83)	3951
CCL20	16	1-16387	0.6648	0.98(0.88-1.09)	3951
CCL21	157	2-21466	0.0002	0.82(0.73-0.91)	3951
CCL22	87	3-2842	0.0002	0.81(0.73-0.91)	3951
CCL23	21	1-763	0.3072	0.94(0.85-1.05)	3951
CCL24	24	2-419	0.1555	0.92(0.83-1.03)	3951
CCL25	34	2-569	2.7e-6	0.77(0.69-0.86)	3951
CCL26	33	1-969	0.1748	0.9(0.77-1.05)	1764
CCL27	35	1-1494	0.0314	0.89(0.8-0.99)	3951
CCL28	174	4-6879	0.1106	0.88()0.75-1.03	1764

Event: relapse-free survival. HR, Hazard radio

Table 3. Prognostic value of CCchemokines expression in breast cancer (Kaplan-Meier Plotter).

Chemokine	Cutoff value expression	Expression	P value	HR	No. Patients
CCL1	10	1-240	0.977	1(0.8-1.24)	1402
CCL2	748	9-18188	0.2119	0.87(0.71-1.08)	1402
CCL3	521	9-13339	0.0186	0.77(0.62-0.96)	1402
CCL4	505	21-10285	0.027	0.72(0.58-0.89)	1402
CCL5	768	55-22269	0.2046	0.87(0.7-1.08)	1402
CCL7	48	1-1374	0.3076	1.12(0.9-1.38)	1402
CCL8	420	10-8024	0.4704	1.08(0.87-1.34)	1402
CCL11	137	3-1956	0.4449	0.92(0.74-1.14)	1402
CCL13	130	4-4102	0.4672	0.92(0.75-1.14)	1402
CCL14	465	3-6969	0.0008	0.69(0.56-0.86)	1402
CCL15	465	3-6969	0.0008	0.69(0.56-0.86)	1402
CCL16	37	1-2126	0.8236	1.02(0.83-1.27)	1402
CCL17	27	2-663	0.022	1.28(1.04-1.59)	1402
CCL18	293	5-23425	0.0156	1.3(1.05-1.61)	1402
CCL19	848	14-15898	0.0015	0.71(0.57-0.88)	1402
CCL20	17	1-16387	0.3747	1.11(0.89-1.37)	1402
CCL21	159	2-21466	0.0031	0.72(0.58-0.9)	1402
CCL22	113	3-2618	0.0183	0.77(0.62-0.96)	1402
CCL23	20	1-763	0.5297	1.07(0.87-1.33)	1402
CCL24	23	2-419	0.007	1.34(1.08-1.66)	1402
CCL25	37	2-569	0.4578	1.08(0.88-1.34)	1402
CCL26	38	1-969	0.6008	1.09(0.8-1.49)	626
CCL27	30	1-704	0.1243	1.18(0.95-1.46)	1402
CCL28	164	4-2786	0.5701	1.11(0.81-1.52)	626

Event: overall survival. HR, Hazard radio

Table4. The Kinase, miRNA and transcription factor-target networks of CCL8 in breast cancer (LinkedOmics).

Enriched Category	Geneset	LeadingEdgeNum	P Value
Kinase Target	Kinase_FYN	7	0
	Kinase_LYN	5	0.008
	Kinase_LCK	4	0.014
	Kinase_CSNK2A1	18	0.017
	Kinase_LCK	1	0.018
miRNA Target	GTCAACC, MIR-380-5P	1	0
	AGCATTA, MIR-155	6	0.051
Transcription Factor Target	V$ETS_Q4	26	0
	V$NFKB_Q6	14	0
	V$COREBINDINGFACTOR_Q6	11	0
	V$NFKAPPAB65_01	16	00
	V$NFKAPPAB_01	17	0

Table5. The Kinase, miRNA and transcription factor-target networks of CCL21 in breast cancer (LinkedOmics).

Enriched Category	Geneset	LeadingEdgeNum	P Value
Kinase Target	Kinase_PLK1	17	0
	Kinase_FYN	11	0
	Kinase_CHEK2	4	0
	Kinase_PRKCA	18	0.004
	Kinase_WEE1	4	0.022
miRNA Target	ATCATGA, MIR-433	12	0
	CCAGGTT, MIR-490	4	0.013
	GGCAGAC, MIR-346	4	0.014
	AGTTCTC, MIR-146A, MIR-146B	3	0.030
	ACAACCT, MIR-453	3	0.031
Transcription Factor Target	V$ETS_Q4	20	0
	V$E2F_02	15	0
	V$E2F1DP1_01	15	0
	V$E2F1DP2_01	15	0
	V$E2F4DP2_01	15	0

Supplementaltable 7. The cox proportional hazard model in CCL8/21 and immune cell infiltration in breast cancer (TIMER)

	coef	HR	95%CI_l	95%CI_u	p.value	sig
Age	0.034	1.034	1.019	1.050	0.000	***
Gender male	0.257	1.293	0.177	9.465	0.800	
Race Black	-0.313	0.731	0.213	2.507	0.618	
Race White	-0.553	0.575	0.177	1.868	0.357	
stage2	0.483	1.622	0.838	3.138	0.151	
stage3	1.325	3.761	1.937	7.305	0.000	***
stage4	2.628	13.848	6.079	31.545	0.000	***
Purity	0.465	1.591	0.549	4.612	0.392	
B_ cell	0.061	1.063	0.010	114.762	0.980	
CD8_T cell	-1.049	0.350	0.021	5.910	0.467	
CD4_T cell	0.881	2.414	0.038	153.508	0.677	
Macrophage	3.135	22.978	1.256	420.509	0.035	*
Neutrophil	0.196	1.217	0.003	581.019	0.950	
Dendritic	-1.025	0.359	0.041	3.153	0.355	
CCL8	0.148	1.159	1.012	1.327	0.033	*
CCL21	0.002	1.002	0.924	1.086	0.961	
